# Current Concepts of Radiographic Evaluation and Surgical Treatment for Hallux Valgus Deformity

**DOI:** 10.3390/jcm14145072

**Published:** 2025-07-17

**Authors:** Byung Ki Cho, Dong Hun Kang, Chan Kang, Gi Soo Lee, Jae Hwang Song

**Affiliations:** 1 Department of Orthopaedic Surgery, College of Medicine, Chungbuk National University, Cheongju 28644, Republic of Korea; titanick25@naver.com; 2 Daejeon Centum Hospital, Daejeon 35209, Republic of Korea; lufid82@naver.com; 3 Department of Orthopaedic Surgery, College of Medicine, Chungnam National University, Daejeon 35015, Republic of Korea; faschan@hanmail.net (C.K.); gs1899@gmail.com (G.S.L.); 4 Department of Orthopaedic Surgery, College of Medicine, Konyang University, Daejeon 35365, Republic of Korea

**Keywords:** hallux valgus, metatarsal osteotomy, open surgery, MIS surgery

## Abstract

Hallux valgus is one of the common causes of forefoot pain in the field of foot and ankle surgery. This condition is characterized by valgus and pronation deformities of the first ray, leading to bunion pain, metatarsalgia, callus formation, and gait disturbances. Conventional open osteotomy of the first metatarsal and proximal phalanx of the first toe has been widely performed. Recently, with increasing reports of favorable radiologic and clinical outcomes of minimally invasive surgery, this technique has been performed by many surgeons. Despite the various surgical methods available, there is still no consensus on the optimal treatment of hallux valgus, and the advantages and disadvantages of open versus minimally invasive techniques remain a topic of debate. This narrative review aims to provide a comprehensive overview of the latest radiographic evaluation and surgical treatment for hallux valgus.

## 1. Introduction

Hallux valgus is one of the most common disorders affecting the first ray. Epidemiological data from the United States indicate that hallux valgus affects approximately 2% to 4% of the general population [1].

Hallux valgus is not merely a lateral deviation of the first toe but rather a complex deformity accompanied by multiple structural abnormalities. The pathomechanics of hallux valgus are driven by an imbalance between the medial and lateral soft tissue structures. When continuous and repetitive external forces are applied to the first toe, the medial soft tissue structures of the first metatarsophalangeal joint (MTPJ) weaken. During this process, the abductor hallucis muscle moves in a plantar direction, leaving the medial joint capsule as the primary supportive structure on the medial aspect of the hallux. Subsequently, the adductor hallucis muscle gradually abducts the first toe due to the weakened resistance and the medial collateral ligament loosens, causing lateral displacement of the sesamoid bones [2]. In simple terms, the contracted lateral soft tissue acts on the first MTPJ much like a bowstring on a bow [3]. The commonly associated deformities include varus deformity of the first metatarsal, valgus deviation of the first toe, medial eminence of the first metatarsal head, pronation of the first toe, and plantar callosity (or corn) beneath the second or third metatarsal [1]. Additionally, hallux valgus is often associated with osteoarthritis of the first MTPJ, claw toe deformities of the lesser toes, and Morton’s neuroma [4].

When conservative treatments fail to provide sufficient relief, surgical correction of hallux valgus becomes necessary. More than 100 surgical techniques have been described for the correction of hallux valgus [5]. Among these, distal chevron osteotomy (DCO), scarf osteotomy, and proximal chevron osteotomy (PCO) are widely accepted conventional procedures, primarily used for correcting distal, shaft, and proximal metatarsal defects, respectively [3,6,7]. For the correction of proximal metatarsal bone, proximal open [8,9] or closing [10] wedge osteotomy is also preferred by some surgeons. Also, the first tarsometatarsal joint (TMTJ) or MTPJ fusion is one of the treatment options for hallux valgus.

Hallux valgus correction can be performed via minimally invasive surgical (MIS) techniques [11,12,13,14]. Recently, MIS techniques have gained popularity due to their advantages, including smaller incisions, reduced soft tissue injury, fewer wound complications, and decreased postoperative pain [12]. There have been several studies reporting the favorable results of moderate and severe hallux valgus treated by MIS techniques [11,12]. However, these techniques still have some concerns. Possible limitations of MIS procedures include metatarsal bone shortening, thermal injuries, and nerve and tendon damage [2,15]. Also some studies have reported that MIS does not improve the radiological and patient satisfaction rate results compared with open surgery [16].

Despite the various surgical methods available, there is still no consensus on the optimal treatment of hallux valgus, and the advantages and disadvantages of open versus minimally invasive techniques remain a topic of debate. Therefore, this review aims to provide a comprehensive overview of the latest radiographic evaluation and surgical treatment for hallux valgus.

## 2. Radiographic Evaluation of Hallux Valgus

### 2.1. X-Ray

**Figure 1:**
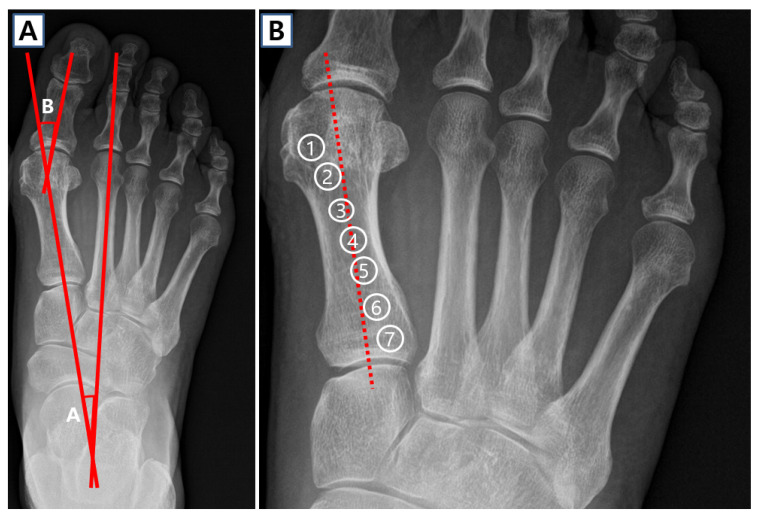
Radiographic parameters of hallux valgus. (**A**) The IMA (angle A) was measured as the angle between a line bisecting the second metatarsal shaft and another line connecting the center of the first metatarsal head to the center of its proximal articular surface. The HVA (angle B) was defined by the angle created at the intersection of the longitudinal axis of the first metatarsal—drawn from the center of its head to the center of its proximal articular surface—and the diaphyseal axis of the proximal phalanx of the great toe. (**B**) The tibial sesamoid position, relative to the longitudinal axis of the first metatarsal, was classified from grade I to VII according to the system described by Hardy and Clapham. IMA: intermetatarsal angle; HVA: hallux valgus angle.

The most critical radiographic view for hallux valgus assessment is the weight-bearing anteroposterior (or dorsoplantar), lateral, and axial sesamoid views, which enable the measurement of various radiographic parameters necessary for classification and surgical decision-making [17]. The key parameters include the hallux valgus angle (HVA), intermetatarsal angle (IMA), tibial sesamoid position, distal metatarsal articular angle (DMAA), MTPJ congruency, and the relative length of the first metatarsal [3]. The tibial sesamoid position is classified into seven grades (I–VII) according to the Hardy and Clapham system (Figure 1) [18]. The presence of a “round sign” evaluates the shape of the lateral edge of the first metatarsal head on anteroposterior radiographs and can also be used to assess the rotation of the first metatarsal [19]. Additionally, the relative length of the first metatarsal in comparison to the second is assessed on anteroposterior radiographs following the method established by Hardy and Clapham [18]. The severity of hallux valgus (mild, moderate, severe) is assessed based on the preoperative IMA and HVA. The moderate group is defined as having first–second IMA between 12 and 16 degrees and a HVA between 20 and 40 degrees. The severe group is defined as first–second IMA greater than 16 degrees or HVA greater than 40 degrees [3]. The sesamoid axial view is important to evaluate the position of the sesamoids in relation to the cristae of the first metatarsal head and to evaluate for subluxation of the sesamoids or rotation of the first metatarsal [17].

### 2.2. Weight-Bearing Computed Tomography

Hallux valgus deformity involves more than just a two-dimensional displacement; it also includes three-dimensional (3D) components, such as inversion and eversion [20,21,22]. A detailed analysis of the 3D aspects of the deformity requires computed tomography (CT) imaging, which enables the reconstruction of 3D models for comprehensive evaluation. However, just as weight-bearing radiographs provide more accurate assessments of hallux valgus compared to non-weight-bearing images, studies have reported that weight-bearing computed tomography (WBCT) is also more precise than non-weight-bearing CT in evaluating the condition [22]. Compared to conventional CT, WBCT has significantly improved the evaluation of complex foot deformities, as it captures images in a physiologic, load-bearing position, enabling a multi-planar assessment of the foot [21,22,23,24,25].

Kim et al. [25] evaluated the first metatarsal pronation angle (α angle) and a sesamoid position (CT 4 position grading) using the semi-weight-bearing coronal CT axial view for the control and hallux valgus groups. On the coronal view, the angle formed by the line that connects the crista and the dorsal center of the first metatarsal and the line vertical to the ground line was defined as the α angle. The sesamoid position was evaluated according to the position of the tibial sesamoid relative to the intersesamoid ridge. A significant difference was noted in the mean α angles between the two groups. From the study, the α angle exceeding 16 degrees was classified as an abnormal first metatarsal pronation angle [25]. There was moderate statistical correlation between the sesamoid position and the HVA. Based on the results, they suggested that there are four different classifications of hallux deformity groups in relation to the α angle (pronation or not) and the sesamoid position (subluxation or not), and the group with pronation and sesamoid subluxation revealed more increased IMA and HVA. Mansur et al. [26] used WBCT to verify the round sign, an indirect sign of first metatarsal pronation in conventional radiographs, and concluded that the round sign weakly correlated with the α angle measured on WBCT.

Kimura et al. [22] evaluated the 3D mobility of each joint of the first ray in feet with hallux valgus compared with normal feet using WBCT and semi-automated 3D software (Mayo Foundation).

The findings of this study indicated that foot loading induces substantial 3D displacement not only at the TMTJ but also in the talonavicular joint, cuneonavicular joint, and MTPJ of the first ray. Patients with hallux valgus exhibited increased mobility in the first ray, including more dorsiflexion, inversion, and adduction of the first TMTJ. A further study by Kimura et al. [21] evaluated the preoperative and postoperative WBCT images of hallux valgus group and demonstrated that hypermobility of the first ray in hallux valgus could be decreased by simply correcting foot alignment without arthrodesis.

Lalevee et al. [27] compared the DMAA in hallux valgus and control populations, before and after computer correction of the first metatarsal pronation and plantarflexion with dedicated WBCT software. This study found that conventional radiograph assessment overestimates the DMAA by 14 degrees, and half of this overestimation was corrected using CT assessment and the other half after pronation correction. They suggested that computerized pronation correction of the first metatarsal bone using WBCT would be needed to objectively evaluate the valgus status of the first metatarsal distal articular surface [27,28].

**Figure 2:**
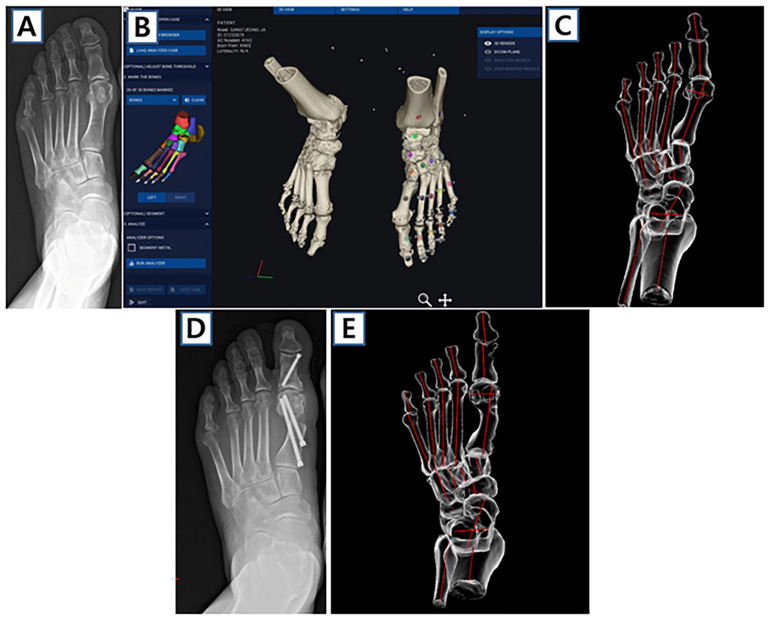
Semi-automatic 3D analysis of hallux valgus deformity using weight-bearing computed tomography and software. (**A**) Preoperative weight-bearing anteroposterior radiograph shows moderate hallux valgus deformity. (**B**) Bone marking with manual registration using the software and (**C**) automated 3D analysis with bone axis. (**D**) Postoperative image after minimally invasive surgery and (**E**) 3D analysis with bone axis.

Recently, sagittal, axial, and coronal bone axes angles in hallux valgus patients were automatically calculated following the manual registration of the bones using WBCT and 3D software (Bonelogic, Helsinki, Finland) (Figure 2) [29]. This study revealed strong associations between metatarsosesamoid complex malposition, coronal pronation of the distal first ray, and conventional axial plane deformities in hallux valgus.

## 3. Operative Treatment of Hallux Valgus

### 3.1. Open Surgery

Numerous surgical techniques have been introduced. However, the fundamental procedures primarily consist of bunionectomy, a distal soft tissue procedure (DSTP) involving lateral soft tissue release and medial imbrication, first metatarsal osteotomy, and proximal phalangeal osteotomy (Akin procedure). Alternatively, in cases of first MTPJ arthritis, arthrodesis may be considered, while first TMTJ instability may warrant a first TMTJ arthrodesis (Lapidus procedure).

**Figure 3:**
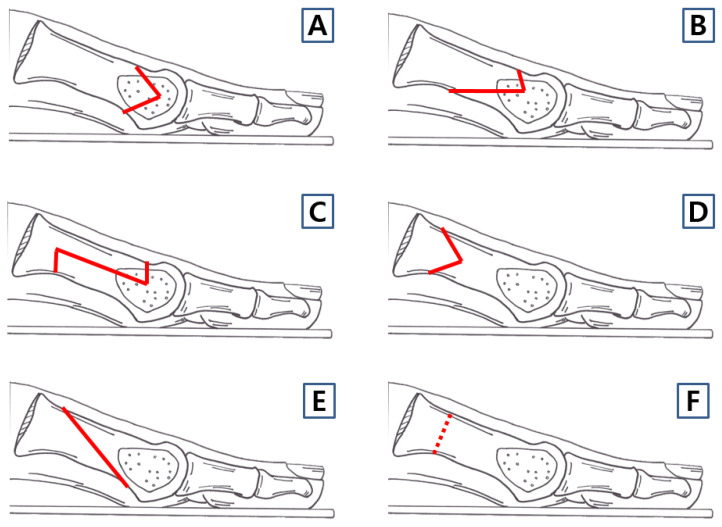
Various surgical techniques of first metatarsal osteotomy for the correction of hallux valgus. (**A**) Distal chevron osteotomy, (**B**) extended distal chevron osteotomy, (**C**) scarf osteotomy, (**D**) proximal chevron osteotomy, (**E**) Ludloff osteotomy, and (**F**) proximal open or close wedge osteotomy.

Metatarsal osteotomy is usually based on the severity of the hallux valgus deformity, categorized into mild, moderate, and severe cases. As the severity of hallux valgus increases, osteotomy of the first metatarsal is performed progressively more proximally rather than distally. The choice of osteotomy technique varies depending on the surgeon’s preference, and numerous studies have extensively reported the advantages and disadvantages of each osteotomy method (Figure 3).

**Figure 4:**
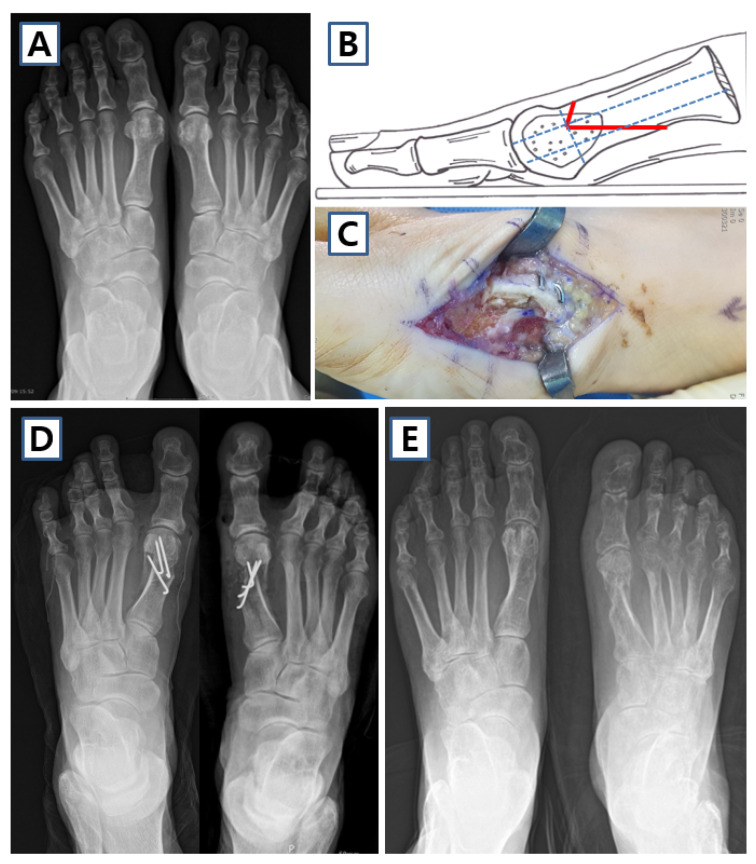
Case of extended distal chevron osteotomy (EDCO) in 59-year-old female. (**A**) Preoperative weight-bearing anteroposterior image shows both moderate hallux valgus deformity. (**B**) Schematic image and (**C**) clinical photo of EDCO. (**D**) Postoperative and (**E**) 1-year follow-up anteroposterior images of the patient.

For mild deformities, correction can be achieved through distal metatarsal osteotomies such as DCO [30] or diaphyseal osteotomies like scarf osteotomy [31,32]. However, certain limitations of DCO persist. Firstly, the restricted contact surface may lead to instability when the capital fragment is shifted laterally [33]. Secondly, there is a risk of osteonecrosis [34], since the plantar osteotomy cut is positioned near the plantar nutrient vessels [35]. Scarf osteotomy is regarded as a technically demanding procedure because of its invasive nature and the risk of troughing, requiring the extended learning curve [7,36]. Recently, several variations in extended distal chevron osteotomies (EDCOs) featuring an elongated plantar limb have been developed to address these issues, demonstrating improved outcomes [3,37]. Song et al. [3] reported the favorable outcomes of EDCO for the treatment of hallux valgus (Figure 4). However, chevron and scarf osteotomies have limitations in terms of correcting pronation deformities [38].

**Figure 5:**
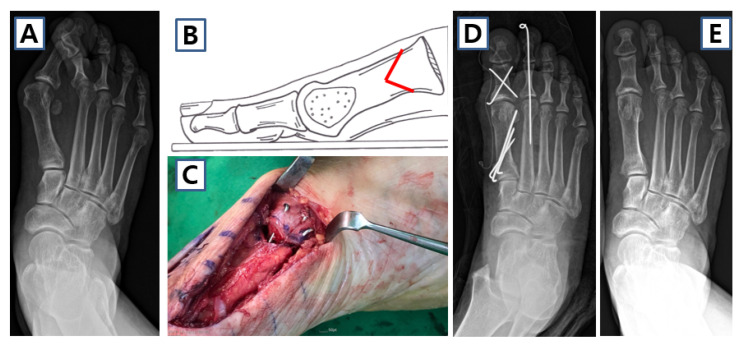
Case of proximal chevron osteotomy (PCO) and Akin osteotomy in 61-year-old female. (**A**) Preoperative weight-bearing anteroposterior image shows right severe hallux valgus deformity. (**B**) Schematic image and (**C**) clinical photo of PCO. (**D**) Postoperative and (**E**) 2-year follow-up anteroposterior images of the patient.

**Figure 6:**
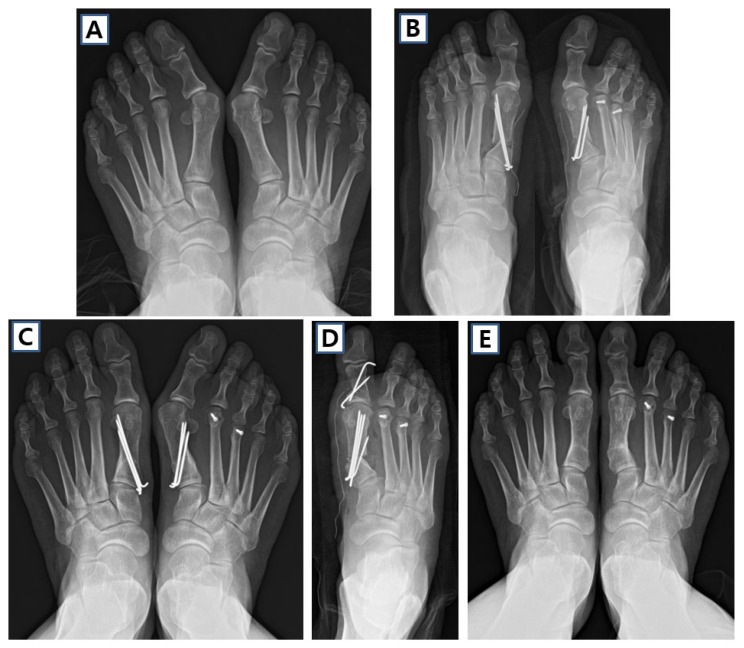
Case of hallux valgus recurrence in 50-year-old female. (**A**) Preoperative weight-bearing anteroposterior image shows severe hallux valgus deformity. (**B**) Postoperative image of both feet after proximal chevron osteotomy (PCO). (**C**) Recurrence of hallux valgus at right foot was shown at 8-month follow-up. (**D**) Revision surgery was performed with PCO and Akin osteotomy. (**E**) Final anteroposterior image of the patients after the implant’s removal.

**Figure 7:**
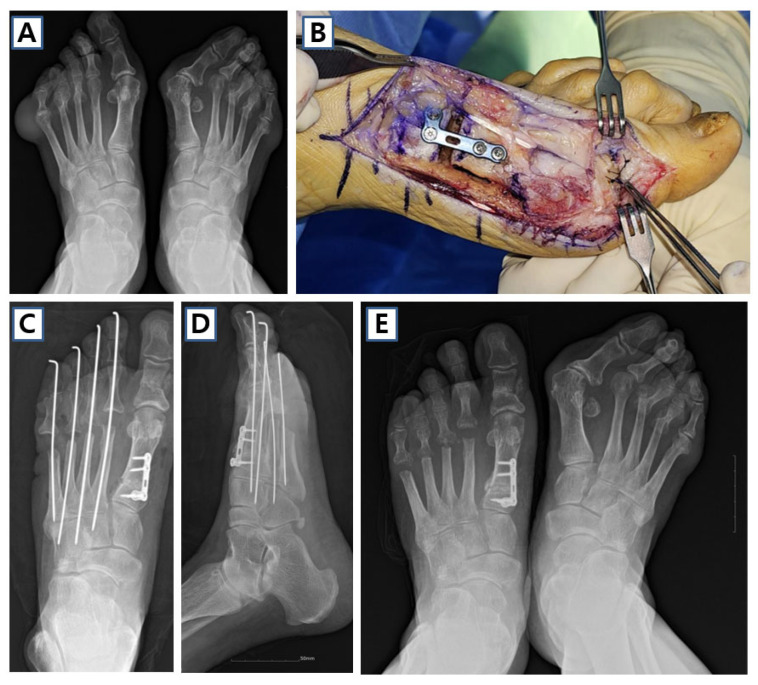
Case of proximal medial opening wedge osteotomy (PMOW) in 63-year-old female. (**A**) Preoperative weight-bearing anteroposterior image shows both severe hallux valgus and rheumatoid feet deformity. (**B**) PMOW and Akin osteotomy was performed on her left first ray, and resection arthroplasty was performed in the lesser metatarsophalangeal joints. (**C**,**D**) Postoperative and (**E**) 3-month follow-up anteroposterior images of the patient.

Moderate deformities may require a combination of DSTP and diaphyseal osteotomy or a proximal metatarsal osteotomy. In cases of severe deformity, DSTP combined with a proximal metatarsal osteotomy or first MTP joint arthrodesis may be necessary. For the correction of proximal metatarsal bone, PCO and open [8,9] or closing [10] wedge osteotomy are commonly performed. PCO is a widely used surgical technique for severe hallux valgus as it provides superior angular correction [39] (Figure 5). However, its drawbacks include a higher complication rate, which stems from the long incision, instability, and an increased risk of transfer metatarsalgia induced by dorsal angulation [40] (Figure 6). Additionally, due to its instability, patients often need longer durations of non-weight-bearing and postoperative rehabilitation. With the recent development of specifically designed, low-profile locking plates for proximal medial opening wedge osteotomy (PMOW), this procedure has gained increasing popularity for correcting moderate-to-severe hallux valgus deformities [41,42,43]. Osteotomy of the procedure is performed at a level 1.5 cm distal to the metatarsocuneiform joint [44] (Figure 7). During PMOW, Han et al. [43] recommended the proximally oriented oblique osteotomy group since oblique osteotomy with a more proximal center of rotation angulation had better results than the straight osteotomy group. Also, other studies recently reported good outcomes with PMOW [42,43,45]. The mean increase in first metatarsal length was reported as 1.2–2.3 mm, and no complications related to lengthening were observed in previous studies.

There have been significant advancements in internal fixation devices for hallux valgus surgery, one of which is the absorbable pin [46]. The protrusion of the metallic implant, such as Kirschner wire (K-wire) or plate, in osteotomy fixation frequently causes skin irritation, and the necessity of a second surgery for its removal poses disadvantages, including additional time, extra costs for the patient, and incision-related pain [46]. Song et al. [46] performed EDCO and Akin osteotomy for the treatment of moderate-to-severe hallux valgus with fixation performed using poly-L-lactic acid (PLLA) pins for the chevron osteotomy and polylactic acid/polyglycolic acid copolymer sutures for the Akin osteotomy. They reported favorable radiological and clinical results of the bioabsorbable implant group comparable with those of the control group treated with metallic implant at 3-year follow-up [46]. In the prospective clinical and experimental study of PLLA pins, a follow-up of more than 5 years is needed to support the results.

### 3.2. Minimally Invasive Surgery

MIS techniques for hallux valgus have recently gained popularity due to their benefits, such as faster recovery, lower pain levels, reduced soft tissue damage, and decreased morbidity [47]. The advancement of MIS techniques in foot surgery has closely mirrored progress in hallux valgus correction [48]. Numerous studies have explored the evolution of surgical techniques and instruments.

The first-generation MIS technique for hallux valgus correction traces back to Isham’s introduction of an incomplete, oblique extra-articular osteotomy of the first metatarsal head [49]. This approach involves dorsomedial closing wedge osteotomy on the first metatarsal head and closing the osteotomy site without fixation. While it offers the benefit of correcting distal metatarsal articular surface deviation, its major limitations include significant metatarsal shortening, osteotomy site instability, and limited effectiveness in correcting the IMA. The second-generation MIS technique involves a transverse osteotomy on the first metatarsal neck without any soft tissue procedures, followed by lateral translation of the metatarsal head and stabilization with a K-wire [50]. However, several studies have highlighted potential risks, such as malunion due to instability and inadequate fixation, recurrence, and surgical site infection [51,52].

The recent application of Shannon burr becomes a key factor in advancements of MIS surgery [48]. Originally utilized by maxillofacial surgeons and dentists for bone surgery, Shannon burrs have since been adopted by foot and ankle surgeons for hallux valgus surgery and have demonstrated versatility in various foot procedures. Available in multiple diameters, lengths, and shapes, they can be selected based on bone type and size, as well as the osteotomy’s shape, location, and direction. Various MIS methods with Shannon burr have been introduced, among which the third-generation MIS, minimally invasive chevron–Akin surgery (MICA) [47], and the fourth-generation MIS, minimally invasive transverse distal metatarsal osteotomy surgery (MITO) [12], are two of the most widely used.

**Figure 8:**
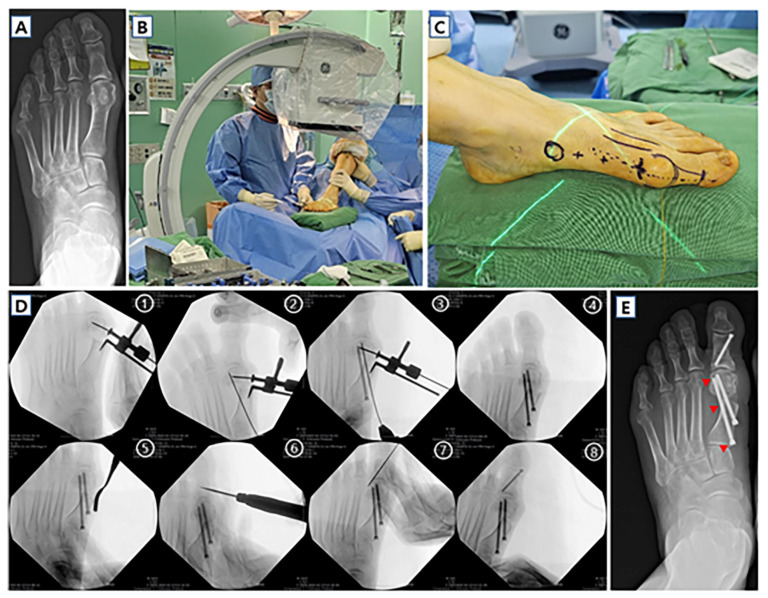
Case of minimally invasive transverse distal metatarsal osteotomy (MITO) in 63-year-old female. (**A**) Preoperative weight-bearing anteroposterior image shows left moderate hallux valgus deformity. Clinical photos of MITO showing (**B**) position of the patient and (**C**) incision sites of the surgery. (**D**) Intraoperative C-arm fluoroscopic images showing the procedures of MITO: (1) lateral translation of the distal fragment using translation guide. (2)–(4) Proximal and distal screws were placed using temporary k-wire fixation. (5) Bumpectomy and ostectomy were performed. (6)–(8) Akin osteotomy was also performed by minimally invasive surgery. (**E**) Two-month follow-up anteroposterior image of the patient. Red triangles indicate 3-point fixation with “in–out–in” proximal screw.

**Figure 9:**
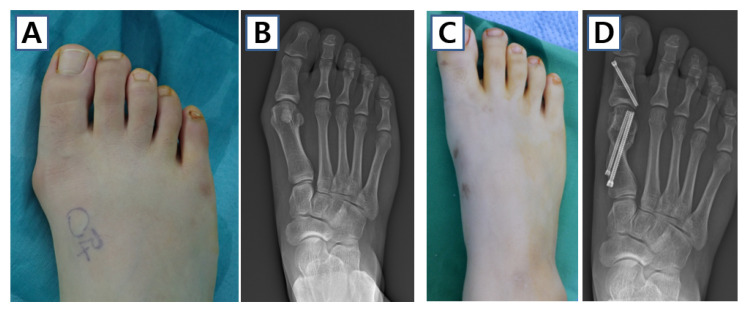
Case of minimally invasive transverse distal metatarsal osteotomy in 27-year-old female. Preoperative (**A**) clinical photo and (**B**) weight-bearing anteroposterior image show right moderate hallux valgus deformity. (**C**) Clinical photo and (**D**) anteroposterior image of the patient at her final follow-up.

Recent studies comparing MIS and open techniques have shown favorable and comparable clinical and radiographic outcomes [53,54]. Both MICA and MITO involve osteotomy on the first metatarsal neck, followed by lateral translation of the metatarsal head and stabilization with headless screws [12]. The placement of the proximal screw with three-point fixation is important, as this allowed for stable fixation of the osteotomy with superior biomechanical property [12,53] (Figure 8 and Figure 9). If joint incongruity of the first MTPJ persists and passive reduction remains limited following metatarsal osteotomy and fixation, a minimally invasive lateral release of the first web space can be performed. The difference between MICA and MITO is that MICA involves performing a chevron osteotomy, whereas MITO involves performing a transverse osteotomy. The authors elaborate on MITO using beveled screw heads and a translation guide as the current standard of care. Beveled screw heads can reduce complications related to screw irritation, and a translation guide can assist the surgeon in performing lateral translation of the metatarsal head more easily.

Yoon et al. [47] reported that the MICA group exhibited lower pain levels on postoperative day 1 and demonstrated better first MTPJ range of motion at 12 months postoperatively. Also, Lewis et al. [14] reported the favorable outcomes of MICA with a recurrence rate of 7.7% and a complication rate of 4.8% in >5 years follow-up. While a chevron osteotomy offers greater structural stability, a transverse osteotomy provides superior translational and rotational control, making it more effective for correcting pronation deformities [12]. MITO is useful for treating pronation deformities and can be strategically employed in cases with poor skin conditions. A cadaveric biomechanical study by Aiyer et al. [55] demonstrated higher ultimate load to failure, yield load, and stiffness with a transverse osteotomy compared to a chevron osteotomy, though the difference was not statistically significant [55]. The authors presumed that chevron osteotomies may result in early failure by relative ease of cutout through cancellous bone compared to transverse osteotomies in which failure requires cortical cutout.

Recently, Yoon et al. [12] reported favorable clinical and radiologic outcomes for patients with mild-to-moderate or severe hallux valgus who underwent MITO with a minimum 24-month follow-up. From this study, up to 100% translation of the metatarsal width with minimal contact between the distal fragment and the proximal fragment was sufficient for optimal recovery. This finding is particularly noteworthy, as a maximum translation of 50% is generally recommended for an open distal chevron osteotomy. They assumed that this can be accomplished using a percutaneous technique, which maintains much of the periosteal sleeve and ensures stability through three-point fixation with “in–out–in” screws.

Choi et al. [56,57] suggested that performing MICA at the proximal level is more advantageous than at the distal level for moderate-to-severe hallux valgus deformities. They reasoned that, much like in open surgery, proximal metatarsal osteotomies offer greater correctional power than those conducted at the distal level.

The postoperative care of MICA and MITO are relatively shorter than open surgery. Patients are permitted to full weight-bearing immediately with hard-soled shoes for the first 4 weeks, with surgical wounds assessed 1 week postoperatively [12].

However, these techniques still have some concerns. Possible drawbacks of these procedures include metatarsal bone shortening caused by the burr’s thickness, inaccuracies in bone cuts, and thermal injuries affecting both the bone and wound edges. Furthermore, there is a risk of damaging proximal nerves, such as the dorsal medial cutaneous nerve, injuries to the metatarsal head’s vascular supply, and harm to the flexor and extensor tendons [2,15]. To diminish these complications, Carvalho et al. [2] found that ensuring safety in the MIS chevron-type osteotomy involves finalizing the dorsal arm at a median distance of 25.6 mm and the plantar arm at 23.9 mm from the most distal point of the first metatarsal head. In a recent study with MITO [12], complications were observed in 20.7% of patients, with major complications occurring in 9.5%. The two most common major complications which required revision surgery were screw irritation and recurrence. Minor complications were reported in 11.2% of patients, with dorsomedial cutaneous nerve irritation being the most frequent, followed by transfer metatarsalgia. A recent meta-analysis comparing MIS and open surgery for hallux valgus found that the risk of hardware removal was significantly higher in the MIS group, likely due to increased screw irritation [58]. However, there was no significant difference in hallux valgus recurrence rates between the two techniques [58,59].

Also, another study reported that MIS showed no advantages over open surgery other than a shorter scar [51]. The stiffness and range of motion of MIS were comparable to those of open surgery. The debate over the effectiveness of MIS versus traditional open techniques for hallux valgus remains unresolved. Also, there is still controversy surrounding the DSTP in MIS. Some recommend lateral release [60,61,62]; others do not view it as an essential component of MIS [63,64]. Hence, further studies are needed to evaluate the effectiveness of hallux valgus deformity correction and the long-term clinical and radiographic outcomes of MIS (Table 1).

### 3.3. Prognosis of Surgery

To date, several studies have investigated the influence of certain factors on clinical and radiological outcomes following hallux valgus surgery. One study reported that age had no impact on perioperative, functional, or subjective outcomes after hallux valgus surgery [65]. However, older patients showed an increased risk of radiological recurrence after surgical correction. Another study reported that the postoperative sesamoid position on anteroposterior standing radiographs was not linked to hallux valgus recurrence according to radiographic criteria [66], and the result contradicted the findings of previous studies [67,68,69]. Cho et al. [70] demonstrated that there was no significant differences in clinical and radiographic outcomes between hallux valgus patients with and without generalized ligamentous laxity, and this condition showed no clear impact on postoperative recurrence of the deformity.

Studies have also been conducted on various factors that negatively affect the postoperative prognosis of hallux valgus. Choi et al. [71] reported that hallux valgus patients with metatarsus adducts had a greater preoperative and postoperative hallux valgus angle, a higher recurrence rate, and lower postoperative satisfaction compared to those without metatarsus adducts after open surgery without lesser metatarsal procedures. The study suggested lesser metatarsal realignment surgery in addition to first ray corrective surgery. Heyes et al. [72] reported that the prevalence of hallux valgus recurrence correlated with the severity of pes planus. The recurrence rate of hallux valgus was 1% in patients with a normal Meary’s angle, 29% in those with Meary’s angle between −4 and −10 degrees, and 47% in cases where Meary’s angle was less than −10 degrees.

Treatments for hallux valgus deformity combined with advanced hallux rigidus or rheumatoid arthritis remain controversial, with fusion surgery being the primary consideration in such cases. However, recent studies have reported that oblique distal osteotomy of the first metatarsal for stage 1–3 hallux rigidus and joint preserving surgery for rheumatoid forefoot deformities revealed favorable radiological and clinical results [73,74]. In cases of hallux valgus with hypermobility of the first ray, proximal osteotomy combined with DSTP (not arthrodesis) showed favorable results, demonstrating a significant reduction in the first ray at the two-year follow-up [75]. These study results contradict previous findings and provide significant implications and new insights for foot and ankle surgeons.

## 4. Conclusions

Despite the advanced diagnostic tools and various surgical methods available, there is still no consensus on the optimal treatment of hallux valgus. When treating hallux valgus, treatment decisions should not be based on a fixed algorithm but rather tailored to each patient’s symptoms and radiographic findings. In particular, during hallux valgus correction, attention should be given not only to the two-dimensional axial plane correction but also to proper 3D multi-plane correction. The use of WBCT is recommended for decision-making and perioperative evaluation in the treatment of hallux valgus. MIS hallux valgus surgery has been increasingly performed in recent years. However, caution is advised against the indiscriminate use of surgery, as concerns remain regarding complications following MIS procedures.

## Figures and Tables

**Table 1:** Comparison of surgical methods for hallux valgus.

Surgical Method	Advantages	Disadvantages	Indications
Distal Chevron Osteotomy	Familiar technique; effective for mild-to-moderate deformities; structurally stable with V-shaped cut.	Limited correction of pronation; risk of osteonecrosis; less translation capacity.	Mild-to-moderate hallux valgus without significant pronation or instability.
Proximal Chevron Osteotomy	Provides greater correction; suitable for severe deformities; relatively simple fixation.	Higher complication rate; longer rehabilitation; risk of dorsal angulation and transfer metatarsalgia.	Severe hallux valgus needing substantial angular correction.
Proximal Medial Opening Wedge Osteotomy	Allows precise angular correction; good for moderate-to-severe deformities; compatible with locking plates.	Risk of instability and recurrence; technically demanding; longer learning curve.	Moderate-to-severe hallux valgus with need for precise correction; useful with locking plate systems.
Minimally Invasive Chevron Akin Osteotomy	Smaller incision; faster recovery; better range of motion; stable fixation with chevron geometry.	Potential for screw irritation; limited ability to correct large deformities; requires specialized equipment.	Mild-to-moderate hallux valgus; when faster recovery and cosmetic outcomes are desired.
Minimally Invasive Transverse Distal Metatarsal Osteotomy	Superior translational and rotational control; effective for pronation correction; good for compromised skin condition.	Higher screw irritation risk; technical challenges; risk of thermal injury and nerve damage.	Moderate-to-severe hallux valgus with pronation; cases requiring minimal soft tissue trauma.

## Data Availability

All data used in this study are included within the article, and additional data are available upon reasonable request from the authors.
